# Trends in the Prevalence and Incidence of Attention-Deficit/Hyperactivity Disorder Among Adults and Children of Different Racial and Ethnic Groups

**DOI:** 10.1001/jamanetworkopen.2019.14344

**Published:** 2019-11-01

**Authors:** Winston Chung, Sheng-Fang Jiang, Diana Paksarian, Aki Nikolaidis, F. Xavier Castellanos, Kathleen R. Merikangas, Michael P. Milham

**Affiliations:** 1 Department of Psychiatry, Kaiser Permanente Northern California, San Francisco; 2 Division of Research, Kaiser Permanente Northern California, Oakland; 3 Genetic Epidemiology Research Branch, National Institute of Mental Health, Bethesda, Maryland; 4 Center for the Developing Brain, Child Mind Institute, New York, New York; 5 Department of Child and Adolescent Psychiatry, Hassenfeld Children’s Hospital at NYU Langone, New York, New York; 6 Center for Biomedical Imaging and Neuromodulation, Nathan S. Kline Institute for Psychiatric Research, Orangeburg, New York

## Abstract

**Importance:**

An increasing prevalence of adult attention-deficit/hyperactivity disorder (ADHD) diagnosis and treatment has been reported in clinical settings and administrative data in the United States. However, there are limited data on recent trends of adult ADHD diagnosis among racial/ethnic subgroups.

**Objective:**

To examine trends, including associated demographic characteristics, psychiatric diagnoses, and negative outcomes, in the prevalence and incidence of adult ADHD diagnosis among 7 racial/ethnic groups during a 10-year period.

**Design, Setting, and Participants:**

This cohort study investigated trends in the diagnosis of ADHD in adults who identified as African American or black, Native American, Pacific Islander, Latino or Hispanic, non-Hispanic white, Asian American, or other using the Kaiser Permanente Northern California health plan medical records. A total of 5 282 877 adult patients and 867 453 children aged 5 to 11 years who received care at Kaiser Permanente Northern California from January 1, 2007, to December 31, 2016, were included. Data analysis was performed from January 2017 through September 2019.

**Exposures:**

Period of ADHD diagnosis.

**Main Outcomes and Measures:**

Prevalence and incidence of licensed mental health clinician–diagnosed ADHD in adults and prevalence of licensed mental health clinician–diagnosed ADHD in children aged 5 to 11 years.

**Results:**

Of 5 282 877 adult patients (1 155 790 [21.9%] aged 25-34 years; 2 667 562 [50.5%] women; 2 204 493 [41.7%] white individuals), 59 371 (1.12%) received diagnoses of ADHD. Prevalence increased from 0.43% in 2007 to 0.96% in 2016. Among 867 453 children aged 5 to 11 years (424 449 [48.9%] girls; 260 236 [30.0%] white individuals), prevalence increased from 2.96% in 2007 to 3.74% in 2016. During the study period, annual adult ADHD prevalence increased for every race/ethnicity, but white individuals consistently had the highest prevalence rates (white individuals: 0.67%-1.42%; black individuals: 0.22%-0.69%; Native American individuals: 0.56%-1.14%; Pacific Islander individuals: 0.11%-0.39%; Hispanic or Latino individuals: 0.25%-0.65%; Asian American individuals: 0.11%-0.35%; individuals from other races/ethnicities: 0.29%-0.71%). Incidence of ADHD diagnosis per 10 000 person-years increased from 9.43 in 2007 to 13.49 in 2016. Younger age (eg, >65 years vs 18-24 years: odds ratio [OR], 0.094; 95% CI, 0.088-0.101; *P* < .001), male sex (women: OR, 0.943; 95% CI, 0.928-0.959; *P* < .001), white race (eg, Asian patients vs white patients: OR, 0.248; 95% CI, 0.240-0.257; *P* < .001), being divorced (OR, 1.131; 95% CI, 1.093-1.171; *P* < .001), being employed (eg, retired vs employed persons: OR, 0.278; 95% CI, 0.267-0.290; *P* < .001), and having a higher median education level (OR, 2.156; 95% CI, 2.062-2.256; *P* < .001) were positively associated with odds of ADHD diagnosis. Having an eating disorder (OR, 5.192; 95% CI, 4.926-5.473; *P* < .001), depressive disorder (OR, 4.118; 95% CI, 4.030-4.207; *P* < .001), bipolar disorder (OR, 4.722; 95% CI, 4.556-4.894; *P* < .001), or anxiety disorder (OR, 2.438; 95% CI, 2.385-2.491; *P* < .001) was associated with higher odds of receiving an ADHD diagnosis. Adults with ADHD had significantly higher odds of frequent health care utilization (OR, 1.303; 95% CI, 1.272-1.334; *P* < .001) and sexually transmitted infections (OR, 1.289; 95% CI 1.251-1.329; *P* < .001) compared with adults with no ADHD diagnosis.

**Conclusions and Relevance:**

This study confirmed the reported increases in rates of ADHD diagnosis among adults, showing substantially lower rates of detection among minority racial/ethnic subgroups in the United States. Higher odds of negative outcomes reflect the economic and personal consequences that substantiate the need to improve assessment and treatment of ADHD in adults.

## Key Points

**Question:**

What are recent trends in adult attention-deficit/hyperactivity disorder (ADHD) prevalence and incidence among racial and ethnic groups in the United States?

**Findings:**

In this cohort study of 5 282 877 patients who identified as African American or black, Native American, Pacific Islander, Latino or Hispanic, non-Hispanic white, Asian American, or other and were cared for at Kaiser Permanente Northern California, the annual adult ADHD prevalence and incidence rates significantly increased over a 10-year period for every race/ethnicity except Native American; rates remained highest among white patients throughout. The presence of other psychiatric diagnoses was found to be a major factor associated with likelihood of adult ADHD diagnosis.

**Meaning:**

The prevalence and incidence of adults diagnosed with ADHD are increasing, although pronounced racial/ethnic inequalities in rates of diagnosis remain.

## Teaser

This cohort study examines trends, including associated demographic characteristics, psychiatric diagnoses, and negative outcomes, in the prevalence and incidence of attention-deficit/hyperactivity disorder (ADHD) diagnosis among 7 racial/ethnic groups in the United States during a 10-year period.

## Introduction

Interest in the phenomenology and continuity of attention-deficit/hyperactivity disorder (ADHD) across the life span has been increasing. Initially motivated by findings of clinical symptoms of ADHD in adults with minimal brain dysfunction,^1^ prospective studies of childhood samples^2,3^ and reports of increased ADHD symptoms in parents of youth with ADHD^4^ have provided converging evidence regarding the continuity of ADHD into adulthood. The growing number of negative outcomes associated with ADHD in adulthood (eg, increased rates of motor vehicle crashes, relationship failure, unemployment, substance use, sexually transmitted infections [STIs], and suicide) highlight the importance of detecting and treating the disorder. However, questions remain about the diagnostic process and true prevalence of ADHD among adults as well as factors that may impede its detection.

Estimates of the prevalence of ADHD in adults have been derived from community and clinical samples. The aggregate prevalence of ADHD in adults in 20 countries from the World Mental Health Surveys^5^ was 2.8%, with greater rates in higher-income countries. Prevalence estimates of ADHD in nationally representative household surveys of adults in the United States were 5.2% in the National Comorbidity Survey Replication^6^ and 2.5% for the combined subtype of ADHD in the National Epidemiologic Survey on Alcohol and Related Conditions.^7^ The consistency in sociodemographic correlates of adult ADHD in the 2 community surveys of nationally representative samples of adults in the United States^6,8^ provides a basis to investigate disparities in the recognition and treatment of ADHD in clinical settings; specifically, male sex, higher education level, divorced marital status, and non-Hispanic white race/ethnicity have been associated with a greater likelihood of diagnosis in 1 or more studies.^6,8^

The cross-sectional nature of household surveys precludes exploration of time trends in the magnitude of adult ADHD. Instead, indirect estimates of ADHD prevalence and time trends in the United States have been derived from administrative databases, including managed care settings,^9,10^ employer health insurance,^11^ Medicaid,^12^ and primary care.^13^ These studies have demonstrated consistent increases in the number of adults in the United States diagnosed with and treated for ADHD. In the largest study of a nationally representative sample of primary care physicians, Fairman et al^13^ reported a 36% increase in the prevalence of ADHD between 2008-2009 and 2012-2013. A parallel increase has been documented in the Scandinavian registries, a setting in which patients have universal health care,^14,15^ so the increase is unlikely to be attributable solely to changes in health insurance coverage.

Despite growing insight into adult ADHD in the general population, it has been difficult to comprehensively characterize trends in the diagnosis and treatment of ADHD among adults because of differences in sampling and methods. Studies differ markedly in design and sample characteristics and have used divergent diagnostic methods and criteria for ADHD, ranging from formal categorical approaches to dimensional symptom checklists.^9^ Additionally, few studies have considered differences in diagnostic trends observed among a broad range of racial/ethnic groups. We evaluated the electronic medical record system of Kaiser Permanente Northern California (KPNC) for the period of January 1, 2007, to December 31, 2016, to quantify trends in the prevalence and incidence of ADHD diagnosis in adults among 7 racial/ethnic groups. Specifically, we tested the following hypotheses: (1) that diagnosis of ADHD in adults is increasing, possibly at a higher rate than among children aged 5 to 11 years, (2) that significant differences in prevalence and incidence by sex and race/ethnicity persist, (3) that diagnosis of ADHD in adults is associated with other diagnosed mental disorders, and (4) that ADHD is negatively associated with selected health outcomes.

## Methods

### Data Source

Kaiser Permanente Northern California is a large, integrated health care delivery system that provides comprehensive medical care to more than 4 million individuals. The population of KPNC members is generally representative of the overall regional population, although income distribution extremes may be underrepresented.^16^ Medical records were used to extract demographic and clinical information, including age, sex, race/ethnicity, marital status, occupational status, medical diagnoses from outpatient and inpatient visits, and pharmacy use. Median family household income and education level were not directly available, so census tract–based median family household income and education level were calculated based on the 2010 US Census and 2006-2010 American Community Survey. The study design was reviewed and approved by the KPNC Region institutional review board, including a waiver of informed consent because the research involved no more than a minimal risk to participants. Our report followed the Strengthening the Reporting of Observational Studies in Epidemiology (STROBE) reporting guideline for cohort studies.

### Study Sample and Measures

The primary study cohort consisted of 5 282 877 adult patients who received care at KPNC between January 1, 2007, and December 31, 2016. We identified ADHD diagnoses from electronic medical records of inpatient and outpatient encounters based on *International Classification of Diseases, Ninth Revision* (*ICD-9*) diagnostic codes (314.x) and *ICD-10* diagnostic codes (F90.x). Diagnosis of ADHD in the KPNC system is typically based on the following: medical history; chief or presenting concerns; mental status examination; and functional ability and structured clinical interview by a qualified, licensed mental health clinician. For those newly diagnosed as adults, childhood onset of symptoms must be reported. In pilot work with 50 participants conducted in preparation for the study, 47 adults (94%) diagnosed with ADHD during the study period were assessed by a licensed psychiatrist, psychologist, or psychiatric nurse practitioner and were subsequently treated with ADHD medications. To facilitate comparison with other databases and to determine the specificity of trends in adults, we also obtained medical records for children aged 5 to 11 years to estimate corresponding prevalences.

Covariates included sex, age, race/ethnicity, census tract–based median family household income and education level, marital status, employment status, health care utilization, and psychiatric and physical comorbidities. Race/ethnicity was self-identified as 1 of the following: African American or black (black), American Indian or Alaska Native (AIAN), Native Hawaiian or other Pacific Islander (NHPI), Latino or Hispanic (Hispanic), non-Hispanic white (white), Asian American (Asian), or other. Psychiatric comorbidities were identified based on diagnostic codes from outpatient and inpatient visits. We calculated a dichotomous variable for whether participants had 1 or more health care visits (inpatient or outpatient) per year during the study period as a measure of health care utilization.^17^

Although not a primary focus, 3 measures of negative associations of ADHD were selected to reflect adverse consequences identified in the adult ADHD literature. First, given prior reports of increased frequency of physical health problems,^18^ we examined health care utilization as defined earlier. Second, motivated by consistent reports of increased accidental injury rates among individuals with ADHD,^19^ we examined whether participants with ADHD were more likely to have frequent emergency department (ED) visits (defined as ≥3 visits to an ED during the 10-year study period documented in the medical record). Finally, findings from a 2018 national longitudinal cohort reaffirmed prior assertions that STIs are more frequent in individuals with ADHD.^20^ Therefore, we used *ICD-9* or *ICD-10* codes to examine differences in the proportion affected by 1 or more STI diagnosis during the study period.

### Statistical Analysis

We estimated annual prevalence rates for diagnosis of ADHD in adults and children by race/ethnicity for each year in the study period (2007-2016). The Cochran-Armitage trend test was performed to demonstrate changes of annual prevalence rates over time by race/ethnicity during the study period.

Univariate and multivariable logistic regression analyses were conducted to estimate odds ratios (ORs) for prevalent ADHD diagnosis by demographic factors, including sex, race/ethnicity, age, area-based median household income, area-based median education level, marital status, employment status, and health care utilization. Multivariable logistic regression models were used to assess the association of other psychiatric diagnoses, occurring at any time during KPNC enrollment, with prevalent ADHD diagnosis. These included depressive disorder, bipolar disorder, substance use disorder, and personality disorder, which are among the most frequent comorbidities in adults with ADHD.^21^ We also included pervasive developmental disorders, eating disorders, and psychotic disorders, which have also been associated with ADHD.^21,22,23,24^ Models containing adjustment for psychiatric diagnoses were assessed for multicollinearity; all variance inflation factors were less than 3.5, indicating that multicollinearity was not a concern. Finally, we examined associations of ADHD diagnosis with negative associations, including frequent ED use, greater health care utilization, and STI diagnosis, using logistic regression with prevalent ADHD diagnosis as the independent variable.

For supplemental analyses, we estimated annual incidence (cases per 10 000 person-years) for diagnosis of ADHD by race/ethnicity for each year in the study period (2007-2016). The Cochran-Armitage trend test was performed to demonstrate changes of annual incidence rates over time by race/ethnicity during the study period. To calculate incidence rate, the *ICD* diagnostic codes were required to be the first on record since enrollment. Members with less than 2 years of continuous enrollment before their index ADHD diagnosis were excluded from incidence calculation. Univariate and multivariable Cox proportional hazard regression analyses were conducted to estimate hazard ratios for developing incident ADHD according to the demographic factors listed earlier as well as other psychiatric diagnoses during KPNC enrollment.

Statistical analyses were performed using SAS version 9.3 (SAS Institute). Analysis was performed from January 2017 through September 2019. *P* < .05 was considered statistically significant, and all tests were 2-tailed. A total of 1 609 723 participants (30.47%) were missing information on census tract income and education, 1 625 722 (30.77%) were missing information on marital status, and 2 845 824 (53.87%) were missing information on employment status, all of which were handled via the missing indicator approach.

## Results

### Prevalence

Table 1 shows the distribution of demographic characteristics and negative outcome in adults based on ADHD status. Among the 5 282 877 adult records (1 155 790 [21.9%] aged 25-34 years; 2 667 562 [50.5%] women; 2 204 493 [41.7%] white individuals) identified in KPNC from January 1, 2007, through December 31, 2016, 59 371 individuals (1.12%) had a diagnosis of ADHD. Prevalence increased from 0.43% in 2007 to 0.96% in 2016. The Figure depicts yearly prevalence rates among adults by racial/ethnic group. During the study period, annual adult ADHD prevalence increased for every race/ethnicity. White individuals consistently had the highest prevalence rates, increasing from 0.67% in 2007 to 1.42% in 2016 compared with increasing 0.11% to 0.35% among Asian individuals, 0.11% to 0.39% among NHPI individuals, 0.22% to 0.69% among black individuals, 0.25% to 0.65% among Hispanic individuals, 0.29% to 0.71% among individuals who identified as other, and 0.56% to 1.14% among AIAN individuals.

**Table 1.** Number of ADHD Cases Among 5 282 877 Adult Members of Kaiser Permanente Northern California, 2007 to 2016

Characteristic	No. (%)		*P* Value
	With No ADHD (n = 5 223 506)	With ADHD (n = 59 371)	
Age at study entry, y			
18-24	1 048 328 (20.07)	17 996 (30.31)	<.001
25-34	1 139 150 (21.81)	16 640 (28.03)	
35-44	946 300 (18.12)	11 186 (18.84)	
45-54	874 395 (16.74)	8389 (14.13)	
55-64	66 2579 (12.68)	4268 (7.19)	
≥65	552 754 (10.58)	892 (1.50)	
Sex			
Male	2 585 069 (49.49)	30 246 (50.94)	<.001
Female	2 638 437 (50.51)	29 125 (49.06)	
Race/ethnicity^a^			
Asian	779 601 (14.92)	3647 (6.14)	<.001
Black	334 753 (6.41)	2353 (3.96)	
Hispanic	898 896 (17.21)	6138 (10.34)	
NHPI	34 363 (0.66)	177 (0.30)	
AIAN	24 627 (0.47)	346 (0.58)	
Other	987 579 (18.91)	5904 (9.94)	
White	2 163 687 (41.42)	40 806 (68.73)	
Census tract median household income, $			
<30 000	151 567 (2.90)	1208 (2.03)	<.001
30 000 to <50 000	677 133 (12.96)	5673 (9.56)	
50 000 to <100 000	2 026 250 (38.79)	22 230 (37.44)	
100 000 to <150 000	672 523 (12.87)	8797 (14.82)	
150 000 to <200 000	94 369 (1.81)	1433 (2.41)	
≥200 000	11 780 (0.23)	191 (0.32)	
Unknown	1 589 884 (30.44)	19 839 (33.42)	
Census tract education level, residents with college degree, %			
<25	1 447 332 (27.71)	11 307 (19.04)	
25 to <50	1 389 654 (26.60)	16 111 (27.14)	
50 to <75	659 245 (12.62)	9799 (16.50)	
≥75	137 713 (2.64)	2320 (3.91)	
Unknown	1 589 562 (30.43)	19 834 (33.41)	
Marital status			
Divorced or separated	208 928 (4.00)	3751 (6.32)	<.001
Married or partnered	1 729 478 (33.11)	17 591 (29.63)	
Single	1 502 085 (28.76)	23 837 (40.15)	
Unknown	1 612 001 (30.86)	13 721 (23.11)	
Widowed	171 014 (3.27)	471 (0.79)	
Employment status			
Employed	1 173 771 (22.47)	18 757 (31.59)	<.001
Retired	546 080 (10.45)	2428 (4.09)	
Student	82 394 (1.58)	1883 (3.17)	
Unemployed	603 246 (11.55)	8494 (14.31)	
Unknown	2 818 015 (53.95)	27 809 (46.84)	
Health care utilization, visits/y			
<1	4 302 052 (82.36)	46 320 (78.02)	<.001
≥1	921 454 (17.64)	13 051 (21.98)	
ED visits/lifetime			
<3	3 775 770 (72.28)	37 438 (63.06)	<.001
≥3	1 447 736 (27.72)	21 933 (36.94)	
STI diagnosis^b^			
Absent	4 997 362 (95.67)	53 871 (90.74)	<.001
Present	226 144 (4.33)	5500 (9.26)	

Abbreviations: ADHD, attention-deficit/hyperactivity disorder; ED, emergency department; STI, sexually transmitted infection.

^a^ Race/ethnicity was self-identified as 1 of the following: African American or black (black), American Indian or Alaska Native (AIAN), Native Hawaiian or other Pacific Islander (NHPI), Latino or Hispanic (Hispanic), non-Hispanic white (white), Asian American (Asian), or other.

^b^ Includes syphilis, chlamydia, human papillomavirus, genital herpes, gonorrhea, and HIV.

**Figure.**
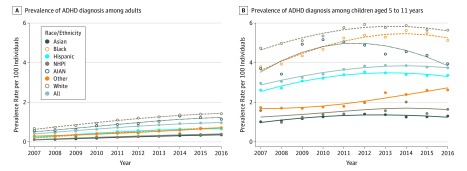
Prevalence Rates of Attention-Deficit/Hyperactivity Disorder (ADHD) Among Adults and Children in the Kaiser Permanente Northern California System, 2007-2016 *Diagnostic and Statistical Manual of Mental Disorders* (Fifth Edition) was published in 2013. Race/ethnicity was self-identified as 1 of the following: African American or black (black), American Indian or Alaska Native (AIAN), Native Hawaiian or other Pacific Islander (NHPI), Latino or Hispanic (Hispanic), non-Hispanic white (white), Asian American (Asian), or other.

To evaluate the extent to which the trends observed were specific to adults and to facilitate comparison with other samples, we examined prevalence in children aged 5 to 11 years treated at KPNC during the same period (Figure). Among the 867 453 children aged 5 to 11 (424 449 [48.9%] girls; 260 236 [30.0%] white individuals), 41 491 (4.78%) had a diagnosis of ADHD. While this prevalence was more than 4-fold greater than that observed in adults, prevalence increased by only 26.4% (ie, from 2.96% in 2007 to 3.74% in 2016) among children compared with the 123.3% increase observed in adults (ie, 0.43% to 0.96%).

Table 2 shows crude and adjusted associations of demographic characteristics with ADHD diagnosis among adults. Odds of diagnosis were lower among all older age groups compared with patients aged 18 to 24 years (eg, patients aged 55-64 years: OR, 0.375; 95% CI, 0.363-0.388; *P* < .001; patients aged >65 years: OR, 0.094; 95% CI, 0.088-0.101; *P* < .001); lower among women than men (OR, 0.943; 95% CI, 0.928-0.959; *P* < .001); lower among members of all nonwhite racial/ethnic groups compared with white patients (eg, Asian patients: OR, 0.248; 95% CI, 0.240-0.257; *P* < .001; NHPI patients: OR, 0.273; 95% CI, 0.236-0.317; *P* < .001); higher among patients who were divorced or separated compared with patients who were single (OR, 1.131; 95% CI, 1.093-1.171; *P* < .001); lower among retired and unemployed persons and those with unknown employment status compared with employed individuals (retired: OR, 0.278; 95% CI, 0.267-0.290; *P* < .001; unemployed: OR, 0.881; 95% CI, 0.859-0.904; *P* < .001; unknown: OR, 0.618; 95% CI, 0.606-0.629; *P* < .001) but higher among students (OR, 1.430; 95% CI, 1.363-1.500; *P* < .001); higher among those living in census tracts with a higher median household income compared with those living in census tracts with a median household income under $30 000 (eg, $150 000 to <$200 000: OR, 1.905; 95% CI, 1.764-2.058; *P* < .001; ≥$200 000: OR, 2.034; 95% CI, 1.744-2.372; *P* < .001); and higher among those living in census tracts with more residents with a college degree compared with those living in census tracts with less than 25% of residents with a college degree (eg, 50% to <75%: OR, 1.903; 95% CI, 1.852-1.955; *P* < .001; ≥75%: OR, 2.156; 95% CI, 2.062-2.256; *P* < .001). These associations were generally similar in direction in the multivariable model, with variation in magnitude. However, after adjustment, being a student was negatively associated with diagnosis (adjusted OR, 0.910; 95% CI, 0.865-0.957; *P* < .001), and odds were slightly lower and the association was not significant for those in census tracts with higher median income levels compared with the lowest category (eg, $150 000 to <$200 000: OR, 0.926; 95% CI, 0.853-1.006; *P* = .07; ≥$200 000: OR, 0.940; 95% CI, 0.802-1.101; *P* = .44).

**Table 2.** Unadjusted and Adjusted ORs of ADHD Diagnosis by Demographic Characteristics and Service Utilization

Characteristic	Unadjusted OR (95% CI)	*P* Value	Adjusted OR (95% CI)^a^	*P* Value
Age at study entry, y				
18-24	1 [Reference]	NA	1 [Reference]	NA
25-34	0.851 (0.833-0.869)	<.001	0.836 (0.816-0.856)	<.001
35-44	0.689 (0.672-0.705)	<.001	0.622 (0.606-0.639)	<.001
45-54	0.559 (0.545-0.574)	<.001	0.413 (0.400-0.425)	<.001
55-64	0.375 (0.363-0.388)	<.001	0.226 (0.217-0.235)	<.001
≥65	0.094 (0.088-0.101)	<.001	0.051 (0.047-0.056)	<.001
Sex				
Male	1 [Reference]	NA	1 [Reference]	NA
Female	0.943 (0.928-0.959)	<.001	0.860 (0.846-0.875)	<.001
Race/ethnicity^b^				
White	1 [Reference]	NA	1 [Reference]	NA
Asian	0.248 (0.240-0.257)	<.001	0.215 (0.208-0.222)	<.001
Black	0.373 (0.357-0.389)	<.001	0.341 (0.327-0.356)	<.001
Hispanic	0.362 (0.352-0.372)	<.001	0.313 (0.304-0.321)	<.001
NHPI	0.273 (0.236-0.317)	<.001	0.247 (0.213-0.286)	<.001
AIAN	0.745 (0.670-0.829)	.04	0.712 (0.640-0.792)	<.001
Other	0.317 (0.308-0.326)	<.001	0.282 (0.274-0.290)	<.001
Marital status				
Single	1 [Reference]	NA	1 [Reference]	NA
Divorced or separated	1.131 (1.093-1.171)	<.001	1.754 (1.689-1.822)	<.001
Married or partnered	0.641 (0.628-0.654)	<.001	0.852 (0.833-0.872)	<.001
Widowed	0.174 (0.158-0.190)	<.001	0.815 (0.739-0.899)	<.001
Unknown	0.536 (0.525-0.548)	<.001	0.725 (0.709-0.742)	<.001
Employment status				
Employed	1 [Reference]	NA	1 [Reference]	NA
Retired	0.278 (0.267-0.290)	<.001	0.944 (0.895-0.994)	.03
Student	1.430 (1.363-1.500)	<.001	0.910 (0.865-0.957)	<.001
Unemployed	0.881 (0.859-0.904)	<.001	0.808 (0.785-0.831)	<.001
Unknown	0.618 (0.606-0.629)	<.001	0.837 (0.819-0.856)	<.001
Census tract, median household income, $				
<30 000	1 [Reference]	NA	1 [Reference]	NA
30 000 to <50 000	1.051 (0.988-1.119)	.12	0.940 (0.883-1.001)	.06
50 000 to <100 000	1.377 (1.299-1.459)	<.001	0.951 (0.896-1.009)	.01
100 000 to <150 000	1.641 (1.545-1.743)	<.001	0.894 (0.839-0.953)	<.001
150 000 to <200 000	1.905 (1.764-2.058)	<.001	0.926 (0.853-1.006)	.07
≥200 000	2.034 (1.744-2.372)	<.001	0.940 (0.802-1.101)	.44
Unknown	1.566 (1.477-1.660)	<.001	1.919 (0.806-4.566)	.14
Census tract education level, residents with college degree, %				
<25	1 [Reference]	NA	1 [Reference]	NA
25 to <50	1.484 (1.449-1.520)	<.001	1.380 (1.345-1.416)	<.001
50 to <75	1.903 (1.852-1.955)	<.001	1.788 (1.732-1.846)	<.001
≥75	2.156 (2.062-2.256)	<.001	2.107 (2.001-2.218)	<.001
Unknown	1.597 (1.561-1.635)	<.001	0.830 (0.349-1.971)	.67
Service utilization, visit/y				
<1	1 [Reference]	NA	1 [Reference]	NA
≥1	1.316 (1.290-1.342)	<.001	1.760 (1.720-1.801)	<.001

Abbreviations: ADHD, attention-deficit/hyperactivity disorder; NA, not applicable; OR, odds ratio.

^a^ Adjusted for all demographic characteristics shown in the table and service utilization.

^b^ Race/ethnicity was self-identified as 1 of the following: African American or black (black), American Indian or Alaska Native (AIAN), Native Hawaiian or other Pacific Islander (NHPI), Latino or Hispanic (Hispanic), non-Hispanic white (white), Asian American (Asian), or other.

Table 3 shows associations of the presence of other psychiatric diagnoses during the study period with ADHD diagnosis. Adjusting for demographic characteristics, all comorbid psychiatric disorder categories were associated with increased odds of ADHD; the strongest associations were observed for eating disorder (OR, 15.869; 95% CI, 15.100-16.677; *P* < .001), bipolar disorder (OR, 8.566; 95% CI, 8.363-8.774; *P* < .001), depressive disorder (OR, 7.788; 95% CI, 7.648-7.932; *P* < .001), personality disorder (OR, 5.947; 95% CI, 5.764-6.135; *P* < .001) and anxiety disorder (OR, 5.139; 95% CI, 5.044-5.236; *P* < .001). When comorbidities were further adjusted (model 2), all associations were reduced in magnitude (eating disorder: OR, 5.192; 95% CI, 4.926-5.473; *P* < .001; depressive disorder: OR, 4.118; 95% CI, 4.030-4.207; *P* < .001; bipolar disorder: OR, 4.722; 95% CI, 4.556-4.894; *P* < .001; anxiety disorder; OR, 2.438; 95% CI, 2.385-2.491; *P* < .001). Psychotic disorders (OR, 0.775; 95% CI, 0.739-0.812; *P* < .001) and personality disorders (OR, 0.477; 95% CI, 0.457-0.499; *P* < .001) were associated with lower odds of diagnosis of ADHD, while pervasive development disorders were not associated with ADHD diagnosis (OR, 0.950; 95% CI, 0.861-1.048; *P* = .31) (Table 3).

**Table 3.** Adjusted ORs of ADHD Diagnosis by Presence of Other Mental Disorder Diagnoses During Study Period

Comorbid Disorder	ADHD Cases, No. (%)	Model 1		Model 2	
		OR (95% CI)^a^	*P* Value	OR (95% CI)^b^	*P* Value
Depressive disorder	33 877 (57.06)	7.788 (7.648-7.932)	<.001	4.118 (4.030-4.207)	<.001
Bipolar disorder	9790 (16.49)	8.566 (8.363-8.774)	<.001	4.722 (4.556-4.894)	<.001
Anxiety disorder	39 075 (65.81)	5.139 (5.044-5.236)	<.001	2.438 (2.385-2.491)	<.001
Psychotic disorder	2355 (3.97)	3.083 (2.952-3.219)	<.001	0.775 (0.739-0.812)	<.001
Personality disorder	5130 (8.64)	5.947 (5.764-6.135)	<.001	0.477 (0.457-0.499)	<.001
Alcohol use disorder	5570 (9.38)	2.925 (2.840-3.011)	<.001	1.108 (1.072-1.146)	<.001
Drug use disorder	9383 (15.80)	2.636 (2.575-2.698)	<.001	1.156 (1.125-1.187)	<.001
Eating disorder	2299 (3.87)	15.869 (15.100-16.677)	<.001	5.192 (4.926-5.473)	<.001
Pervasive developmental disorder	488 (0.82)	2.236 (2.038-2.454)	<.001	0.950 (0.861-1.048)	.31

Abbreviations: ADHD, attention-deficit/hyperactivity disorder; OR, odds ratio.

^a^ Compared with absence of disorder and adjusted for age at study entry, sex, race/ethnicity, marital status, employment status, and census tract median household income and education level.

^b^ Compared with absence of disorder and adjusted for age at study entry, sex, race/ethnicity, marital status, employment status, census tract median household income and education level, and all other mental disorders.

Table 4 shows ORs for ED visits, health care utilization, and STI diagnoses during the study period among adults with and without ADHD diagnoses. Adjusting for demographic characteristics only, those with ADHD diagnoses had higher odds of all 3 outcomes (ED visits: OR, 1.752; 95% CI, 1.718-1.787; *P* < .001; health care utilization: OR, 1.812; 95% CI, 1.770-1.854; *P* < .001; STI diagnosis: 1.766; 95% CI, 1.715-1.818; *P* < .001). After additional adjustment for psychiatric comorbidities, all 3 associations were still statistically significant but reduced in magnitude. Those with ADHD still exhibited greater health care utilization (OR, 1.303; 95% CI, 1.272-1.334; *P* < .001) and higher odds of STI diagnosis (OR, 1.289; 95% CI, 1.251-1.329; *P* < .001) but had lower odds of ED visits (OR, 0.911; 95% CI, 0.892-0.930; *P* < .001).

**Table 4.** Associations of ADHD Diagnosis With Emergency Department Visits, Health Service Utilization, and STI Diagnosis

Outcome	ADHD, No. (%)		Model 1		Model 2	
	Yes	No	OR (95% CI)^a^	*P* Value	OR (95% CI)^b^	*P* Value
Emergency department visits	21 933 (36.94)	1 447 736 (27.72)	1.752 (1.718-1.787)	<.001	0.911 (0.892-0.930)	<.001
Service utilization^c^	13 051 (21.98)	921 454 (17.64)	1.812 (1.770-1.854)	<.001	1.303 (1.272-1.334)	<.001
STI diagnosis^d^	5500 (9.26)	226 144 (4.33)	1.766 (1.715-1.818)	<.001	1.289 (1.251-1.329)	<.001

Abbreviations: ADHD, attention-deficit/hyperactivity disorder; OR, odds ratio; STI, sexually transmitted infection.

^a^ Adjusted for age at study entry, sex, race/ethnicity, marital status, employment status, and census tract median household income and education level.

^b^ Adjusted for age at study entry, sex, race/ethnicity, marital status, employment status, census tract median household income and education level, and psychiatric comorbidities, including depressive, bipolar, anxiety, psychotic, personality, alcohol use, drug use, eating, and pervasive developmental disorders.

^c^ Defined as at least 1 visit per year during the study period.

^d^ Includes syphilis, chlamydia, human papillomavirus, genital herpes, gonorrhea, and HIV.

### Incidence

The number of incident cases of ADHD and person-years of observation among the 5 282 877 adult KPNC members identified from January 1, 2007, through December 31, 2016, according to demographic characteristics and negative outcomes, appear in eTable 1 in the Supplement. The overall rate of annual adult ADHD incidence per 10 000 person-years increased from 9.43 in 2007 to 13.49 in 2016. The eFigure in the Supplement shows yearly incidence rates among different racial/ethnic groups. During the study period, a statistically significant increase in adult ADHD incidence occurred in every racial/ethnic group except among AIAN individuals. White persons consistently had the highest incidence rates throughout the study period, increasing from 14.28 per 10 000 person-years in 2007 to 18.42 in 2016. Differences between white individuals and members of other races/ethnicities decreased somewhat over time, most notably among black and Asian persons, who were among those with the lowest incidences in 2007. Specifically, from 2007 to 2016, the annual adult ADHD incidence rate per 10 000 person-years increased from 3.23 to 6.88 among Asian individuals, from 4.67 to 7.54 among NHPI individuals, from 4.68 to 10.62 among black individuals, from 6.22 to 10.76 among Hispanic individuals, from 6.18 to 9.94 among members of other racial/ethnic groups, and from 9.90 to 13.16 among AIAN individuals. Increases over time were slightly curvilinear in all racial/ethnic groups; among AIAN individuals, who constituted the smallest group, the trend was highly curvilinear and peaked in 2014 (eFigure in the Supplement).

Using incident cases of ADHD diagnosis, eTable 2, eTable 3, and eTable 4 in the Supplement reflect generally similar associations as those seen using prevalence data. An exception was that the highest incidence of ADHD diagnosis was seen in students and those unemployed compared with those employed (students: adjusted OR, 1.656; 95% CI, 1.564-1.753; *P* < .001; unemployed persons: adjusted OR, 1.323; 95% CI, 1.280-1.368; *P* < .001).

## Discussion

The increase in rates of ADHD in adults in our sample confirms the results of several other large studies of both community and primary care samples in US and Scandinavian registries.^12,13,14,25^ This global increase in rates of ADHD cannot be attributed to country-specific insurance coverage or health care service systems. Rather, it could reflect increasing recognition of ADHD in adults by physicians and other clinicians as well as growing public awareness of ADHD during the decade under study. The lack of increase in ADHD in youth in our sample is consistent with results from a 2014 meta-analysis.^26^ Our data cannot address whether the trends in our study reflect valid diagnoses, increased treatment seeking for ADHD among adults, and/or increased recognition by clinicians in the KPNC system. There are many challenges to diagnosing ADHD in adults compared with the well-established definitions and assessments in youth.

White adults in this study consistently had the highest rates of ADHD throughout the study period, while Asian and NHPI adults had the lowest rates. This pattern was especially apparent for adult ADHD prevalence (Figure) and is consistent with recent national data on clinician diagnoses^13^ and with estimates of clinician diagnosis in the National Comorbidity Survey Replication.^6^ Similar findings show lower rates of mood disorders in racial/ethnic minority adults in several US community surveys.^27^

A possibility is that the greater prevalence of ADHD in white adults could be a true finding.^28^ The pattern we observed in adults mimics our findings in children aged 5 to 11 years in KPNC as well as those of a prior study of children of the same age enrolled in Kaiser Permanente of Southern California,^29^ which reported that ADHD prevalence in Asian and Pacific Islander patients (ie, 1.1%) was low compared with white patients (ie, 4.5%). However, the lower rates of ADHD in black adults in our sample were not evident in black youth, in whom ADHD prevalence approached that of white individuals by the end of the study period.

Racial/ethnic differences could also reflect differential rates of treatment seeking or access to care. Because this study was conducted using KPNC data, differences in treatment access related to insurance status should be obviated, although other enabling resources may be unequally distributed.^30^ Racial/ethnic background is known to play an important role in opinions on mental health services, health care utilization,^31^ and physician preferences.^32^ In addition, rates of diagnosis-seeking to obtain stimulant medication for nonmedical use may be more common among white vs nonwhite patients.^33^ Ethnic disparities could also be caused by differences in detection or attribution of ADHD symptoms in racial/ethnic minorities, although evidence for this type of bias is mixed.^34,35,36^ Finally, cultural influences on the manifestation or expression of ADHD could lead to racial/ethnic variation in rates.^37^

Pharmacological cognitive enhancement with prescription and illegal stimulants among individuals not diagnosed with ADHD has been noted to be increasing.^38^ Our findings of increasing risk of ADHD diagnosis in those living in census tracts with higher median levels of education and of the highest risk of ADHD diagnosis in those identified as students may reflect that some individuals are seeking diagnosis and treatment for purposes of cognitive enhancement.

Our results regarding comorbidity with ADHD across all diagnostic categories were consistent with patterns of comorbidity in large community-based samples. In the World Mental Health Surveys,^5^ 17.7% of adults with ADHD also met criteria for 3 or more other classes of mental disorders. This highlights the importance of ADHD as a multisystem disturbance that requires comprehensive assessment, irrespective of the primary condition that led to treatment entry. Treatment of ADHD in the context of comorbid conditions also requires further consideration. Use of stimulant medications without recognition of comorbid bipolar disorder or anxiety could lead to their exacerbation. Further, our finding of increased STI diagnosis among those with ADHD, even after adjustment for comorbidities, confirmed the findings of a 2018 population-based study in Taiwan^20^ and has clear preventive implications.

A major challenge in the diagnosis of ADHD in adults is that the assessment typically relies on retrospective informant self-report. Additionally, when assessed in youth, ADHD is often the primary condition, whereas in adults, comorbid illnesses can obfuscate the attribution of symptoms to ADHD. Moreover, consequences of ADHD (eg, substance abuse or behavioral problems) can also complicate the clinical picture. This highlights the need for comprehensive assessment of adults with ADHD that focuses on the overlap of its core features with those of other conditions as well as a detailed history of the evolution and consequences of ADHD. Another distinction between adult and childhood ADHD lies in the pathways to identification. Whereas adults may recognize their own symptoms and seek treatment, youth are more likely to undergo evaluation because of recognition by a parent or teacher. Adult vs child ADHD diagnoses also differ in symptom thresholds (5 vs 6) that might affect diagnostic sensitivity and specificity in delineating ADHD from other forms of illness.

### Limitations

While the scale of the KPNC sample used in the present work is unprecedented, a key limitation is that it is derived from a single health care system in a specific US region. Of note, prior comparisons of residents of Northern California who participate in the KPNC system with those who do not found them highly comparable, although a lower percentage of non-Hispanic white individuals and very low-income participants were present in the KPNC system.^16^ However, the consistency of our findings with those from other regions, countries, and health care systems increased confidence in their generalizability. As noted earlier, because this is a study of administrative incidence and prevalence, we were unable to distinguish true differences in disorder rates from differences in rates of treatment. Population-based longitudinal studies of ADHD in adults would be helpful in addressing this issue.

## Conclusions

Despite these limitations, we confirmed increasing rates of ADHD diagnosis among adults over a 10-year period, albeit with substantially lower rates of detection among the major racial/ethnic subgroups in our sample. Irrespective of the explanations for racial/ethnic differences in ADHD diagnoses, our findings suggest 2 important future efforts to bridge gaps in recognition, diagnosis, and treatment of ADHD in racial/ethnic subgroups. First, there should be an increased focus on careful, unbiased, structured screening and documentation of symptoms across development, especially as the field attempts to further delineate the temporal precedence of ADHD, patterns of comorbidity, and its consequences. Second, greater consideration must be placed on cultural influences on health care seeking and delivery, along with an increased understanding of the various social, psychological, and biological differences among races/ethnicities as well as culturally sensitive approaches to identify and treat ADHD in the total population.

## Notes

Supplement. **eTable 1. **Number of Incident ADHD Cases in Adults and Person-Years of Observation **eTable 2. **Unadjusted and Adjusted Hazard Ratios (HRs) of Incident ADHD Diagnosis in Adults by Demographic Characteristics and Service Utilization **eTable 3. **Adjusted HRs of Incident ADHD Diagnosis in Adults by the Presence of Other Mental Disorder Diagnoses **eTable 4. **Associations of ADHD Diagnosis in Adults With Emergency Department Visits, Health Service Utilization, and Sexually Transmitted Infections **eFigure. **Adult ADHD Incidence by Year by Race/Ethnicity
