# Supplementary material for: Trends in the Prevalence and Incidence of Attention-Deficit/Hyperactivity Disorder Among Adults and Children of Different Racial and Ethnic Groups
Source: JAMA Netw Open. 2019 Nov 1;2(11):e1914344. doi: 10.1001/jamanetworkopen.2019.14344 (PMC6826640; doi:10.1001/jamanetworkopen.2019.14344)
Supplement: Supplement. — eTable 1. Number of Incident ADHD Cases in Adults and Person-Years of Observation eTable 2. Unadjusted and Adjusted Hazard Ratios (HRs) of Incident ADHD Diagnosis in Adults by Demographic Characteristics and Service Utilization eTable 3. Adjusted HRs of Incident ADHD Diagnosis in Adults by the Presence of Other Mental Disorder Diagnoses eTable 4. Associations of ADHD Diagnosis in Adults With Emergency Department Visits, Health Service Utilization, and Sexually Transmitted Infections eFigure. Adult ADHD Incidence by Year by Race/Ethnicity [file jamanetwopen-2-e1914344-s001.pdf]

Supplementary Online Content

Chung W, Jiang S-F, Paksarian D, et al. Trends in the prevalence and incidence of attention-deficit/hyperactivity disorder among adults and children of different racial and ethnic groups. *JAMA Netw Open.* 2019;2(11):e1914344. doi:10.1001/jamanetworkopen.2019.14344

eTable 1. Number of Incident ADHD Cases in Adults and Person-Years of Observation

eTable 2. Unadjusted and Adjusted Hazard Ratios (HRs) of Incident ADHD Diagnosis in Adults by Demographic Characteristics and Service Utilization

eTable 3. Adjusted HRs of Incident ADHD Diagnosis in Adults by the Presence of Other Mental Disorder Diagnoses

eTable 4. Associations of ADHD Diagnosis in Adults With Emergency Department Visits, Health Service Utilization, and Sexually Transmitted Infections

eFigure. Adult ADHD Incidence by Year by Race/Ethnicity

This supplementary material has been provided by the authors to give readers additional information about their work.

© 2019 Chung W et al. *JAMA Network Open.*

eTable 1. Number of Incident ADHD Cases in Adults and Person-Years of Observation

Among 5,282,877 members of Kaiser Permanente Northern California aged 18 years and older, followed from 2007–2016

**Characteristic**	**Cases (N,%)**<br>N=33274	**Person Years**<br>(n=24950869)
**Age at study entry in years**		
18-24	12809(38.50)	4,597,552
25-34	7809(23.47)	4,133,207
35-44	6357(19.11)	4,370,240
45-54	4193(12.60)	4,651,577
55-64	1747(5.25)	3,791,963
65+	359(1.08)	3,406,331
**Sex**		
Male	16759(50.37)	11,871,089
Female	16515(49.63)	13,079,780
**Race/Ethnicity**		

© 2019 Chung W et al. *JAMA Network Open.*

Asian	2453(7.37)	4,021,230
Black	1648(4.95)	1,691,419
Hispanic	4225(12.70)	4,227,753
Islander	115(0.35)	157,290
Native	210(0.63)	116,127
Other	2806(8.43)	2,711,834
White	21817(65.57)	12,025,217
**Marital Status**		
Divorced/Separated	2121(6.37)	1,279,404
Married/Partnered	10295(30.94)	10,589,395
Single	13945(41.91)	6,118,011
Widowed	274(0.82)	1,192,210
Unknown	6639(19.95)	5,771,850
**Employment Status**		

© 2019 Chung W et al. *JAMA Network Open.*

Employed	11591(34.84)	7,336,050
Retired	1185(3.56)	4,130,781
Student	1522(4.57)	294,055
Unemployed	6124(18.40)	2,487,725
Unknown	12852(38.62)	10,702,258
**Census Tract Median Household Income**		
<$30,000	882(2.65)	694,316
$30,000-<$50,000	4102(12.33)	3,377,497
$50,000-<$100,000	16159(48.56)	11,344,558
$100,000-<$150,000	6342(19.06)	4,120,285
$150,000-<$200,000	1034(3.11)	602,061
>=$200,000	140(0.42)	77,372
Unknown	4615(13.87)	4,734,780
**Census Tract Education Level**		

© 2019 Chung W et al. *JAMA Network Open.*

<25% with college degree	8454(25.41)	7,391,237
25-<50% with college degree	11762(35.35)	8,007,365
50-<75% with college degree	6899(20.73)	3,985,822
>=75% with college degree	1549(4.66)	833,193
Unknown	4610(13.85)	4,733,253
**Service Utilization**		
<1 Visit/Year	24096(72.42)	16,474,151
>=1 Visit/Year	9178(27.58)	8,476,718
**Emergency Room Visits**		
<3 Visits	18329(55.09)	15,890,022
>=3 Visits	14945(44.91)	9,060,847
**Sexually Transmitted Infection**		
Absent	29801(89.56)	23,630,641
Present	3473(10.44)	1,320,228

© 2019 Chung W et al. *JAMA Network Open.*

Note: ADHD = attention-deficit/hyperactivity disorder; sexually transmitted infections include syphilis, chlamydia, human papilloma virus, genital herpes, gonorrhea and human immunodeficiency virus.

© 2019 Chung W et al. *JAMA Network Open.*

eTable 2. Unadjusted and Adjusted Hazard Ratios (HRs) of Incident ADHD Diagnosis in Adults by Demographic Characteristics and Service Utilization

Among 5,282,877 members of Kaiser Permanente Northern California aged 18 years and older followed from 2007–2016

Characteristics	Unadjusted HR (95% CI)	P value	Adjusted HR (95% CI)	P value
**Age at study entry in years**				
18-24	1.00 (ref)		1.00 (ref)	
25-34	0.683(0.664-0.702)	<.001	0.790(0.765-0.815)	<.001
35-44	0.495(0.480-0.510)	<.001	0.530(0.512-0.549)	<.001
45-54	0.298(0.288-0.309)	<.001	0.287(0.276-0.299)	<.001
55-64	0.151(0.143-0.158)	<.001	0.135(0.127-0.143)	<.001
65+	0.034(0.031-0.038)	<.001	0.030(0.027-0.034)	<.001
**Sex**				
Male	1.00 (ref)		1.00 (ref)	

© 2019 Chung W et al. *JAMA Network Open.*

female	0.911 (0.892-0.931)	<.001	0.917(0.896-0.937)	<.001
**Race/Ethnicity**				
White	1.00 (ref)		1.00 (ref)	
Asian	0.340(0.326-0.354)	<.001	0.285(0.273-0.297)	<.001
Black	0.541(0.515-0.569)	<.001	0.438(0.416-0.461)	<.001
Hispanic	0.562(0.544-0.581)	<.001	0.433(0.419-0.448)	<.001
Islander	0.414(0.345-0.498)	<.001	0.329(0.274-0.396)	<.001
Native	1.017(0.887-1.165)	0.812	0.869(0.759-0.996)	0.043
Other	0.626(0.602-0.651)	<.001	0.524(0.503-0.545)	<.001
**Marital Status**				
Single	1.00 (ref)		1.00 (ref)	

© 2019 Chung W et al. *JAMA Network Open.*

Divorced/Separated	0.675(0.645-0.707)	<.001	1.520(1.447-1.597)	<.001
Married/Partner	0.395(0.385-0.405)	<.001	0.685(0.665-0.706)	<.001
Widowed	0.092(0.082-0.104)	<.001	0.798(0.702-0.907)	0.001
Unknown	0.508(0.493-0.523)	<.001	0.688(0.666-0.710)	<.001
**Employment Status**				
Employed	1.00 (ref)		1.00 (ref)	
Retired	0.176(0.166-0.187)	<.001	0.896(0.832-0.965)	0.004
Student	3.665(3.473-3.867)	<.001	1.656(1.564-1.753)	<.001
Unemployed	1.673(1.622-1.726)	<.001	1.323(1.280-1.368)	<.001
Unknown	0.818(0.797-0.839)	<.001	0.895(0.869-0.922)	<.001
**Census Tract Median Household Income**				

© 2019 Chung W et al. *JAMA Network Open.*

<$30,000	1.00 (ref)		1.00 (ref)	
$30,000-<$50,000	0.946(0.880-1.017)	0.135	0.907(0.844-0.976)	0.009
$50,000-<$100,000	1.095(1.024-1.172)	0.008	0.899(0.839-0.963)	0.002
$100,000-<$150,000	1.172(1.093-1.258)	<.001	0.840(0.780-0.904)	<.001
$150,000-<$200,000	1.303(1.191-1.426)	<.001	0.888(0.807-0.978)	0.015
>=$200,000	1.369(1.146-1.636)	0.001	0.919(0.765-1.103)	0.362
Unknown	0.835(0.776-0.897)	<.001	2.541(1.065-6.063)	0.036
**Census Tract Education Level**				
<25% with college degree	1.00 (ref)		1.00 (ref)	
25-<50% with college degree	1.267(1.233-1.303)	<.001	1.311(1.272-1.351)	<.001
50-<75% with college degree	1.486(1.440-1.534)	<.001	1.629(1.569-1.691)	<.001

© 2019 Chung W et al. *JAMA Network Open.*

>=75% with college degree	1.597(1.513-1.686)	<.001	1.824(1.715-1.939)	<.001
Unknown	0.937(0.904-0.972)	0.001	0.254(0.107-0.606)	0.002
**Service Utilization**				
<1 visit/year	1.00 (ref)		1.00 (ref)	
>=1 visit/year	0.639(0.624-0.655)	<.001	1.003(0.976-1.031)	0.820

Note: ADHD = attention-deficit/hyperactivity disorder; HR = hazard ratio; CI = confidence interval. Adjusted HRs are adjusted for all demographics shown in the table and service utilization.

© 2019 Chung W et al. *JAMA Network Open.*

eTable 3. Adjusted HRs of Incident ADHD Diagnosis in Adults by the Presence of Other Mental Disorder Diagnoses

During the study period among 5,282,877 members of Kaiser Permanente Northern California aged 18 years and older and followed from 2007–2016

**Comorbid Disorder**		**ADHD Cases (N,%)**	**Person-years**	**Model 1 HR (95% CI)**	**P value**	**Model 2 HR (95% CI)**	**P value**
Depressive Disorder	No	13106(39.39)	19,737,734	1.00 (ref)		1.00 (ref)	
	Yes	20168(60.61)	5,213,135	6.062(5.921-6.207)	<.001	3.457(3.363-3.554)	<.001
Bipolar Disorder	No	27896(83.84)	24,341,332	1.00 (ref)		1.00 (ref)	
	Yes	5378(16.16)	609,537	5.385(5.224-5.551)	<.001	2.900(2.778-3.028)	<.001
Anxiety Disorder	No	9564(28.74)	15,882,360	1.00 (ref)		1.00 (ref)	
	Yes	23710(71.26)	9,068,510	4.508(4.396-4.623)	<.001	2.305(2.239-2.373)	<.001
Psychotic Disorder	No	31886(95.83)	24,429,818	1.00 (ref)		1.00 (ref)	

© 2019 Chung W et al. *JAMA Network Open.*

	Yes	1388(4.17)	521,051	2.305(2.182-2.434)	<.001	0.728(0.687-0.771)	<.001
Personality Disorder	No	30426(91.44)	24,513,806	1.00 (ref)		1.00 (ref)	
	Yes	2848(8.56)	437,064	3.903(3.752-4.061)	<.001	0.564(0.535-0.595)	<.001
Alcohol Use Disorder	No	29896(89.85)	24,035,887	1.00 (ref)		1.00 (ref)	
	Yes	3378(10.15)	914,982	2.624(2.530-2.721)	<.001	1.144(1.099-1.191)	<.001
Drug Use Disorder	No	27628(83.03)	23,271,888	1.00 (ref)		1.00 (ref)	
	Yes	5646(16.97)	1,678,981	2.347(2.279-2.417)	<.001	1.115(1.078-1.152)	<.001
Eating Disorder	No	31757(95.44)	24,895,962	1.00 (ref)		1.00 (ref)	
	Yes	1517(4.56)	54,907	10.579(10.035-11.153)	<.001	3.934(3.727-4.152)	<.001

© 2019 Chung W et al. *JAMA Network Open.*

Pervasive Developmental	No	33035(99.28)	24,898,950	1.00 (ref)		1.00 (ref)	
	Yes	239(0.72)	51,920	1.244(1.094-1.414)	0.001	0.619(0.544-0.703)	<.001

Note: ADHD = attention-deficit/hyperactivity disorder; HR = hazard ratio; CI = confidence interval; Model 1 is adjusted for age at study entry, sex, race/ethnicity, marital status, employment status, and census tract median household income and education level. Model 2 is additionally adjusted for all other mental disorders.

© 2019 Chung W et al. *JAMA Network Open.*

eTable 4. Associations of ADHD Diagnosis in Adults with Emergency Department Visits, Health Service Utilization, and Sexually Transmitted Infections

During the study period among 5,282,877 members of Kaiser Permanente Northern California aged 18 years and older and followed from 2007-2016

	**ADHD Yes(N,%)**	**ADHD No(N,%)**	**Model 1 OR (95% CI)**	**P value**	**Model 2 OR (95% CI)**	**P value**
Emergency Room Visits	14945(44.91)	1454724(27.71)	1.791(1.748-1.836)	<.001	0.957(0.933-0.983)	<.001
Service Utilization	9178(27.58)	925327(17.63)	1.870(1.819-1.922)	<.001	1.357(1.319-1.396)	<.001
STI	3473(10.44)	228171(4.35)	1.829(1.763-1.897)	<.001	1.327(1.278-1.377)	<.001

Note: OR = odds ratio; CI = confidence interval; ADHD = attention-deficit/hyperactivity disorder; STI = sexually transmitted infection. Service utilization defined as at least one visit per year during the study period. Model 1 adjusted for age at study entry, sex, race/ethnicity, marital status, employment status, and census tract median household income and education level. Model 2 additionally adjusted for psychiatric comorbidities, including depressive, bipolar, anxiety, psychotic, personality, alcohol use, drug use, eating and pervasive developmental disorders.

© 2019 Chung W et al. *JAMA Network Open.*

eFigure. Adult ADHD Incidence by Year by Race/Ethnicity

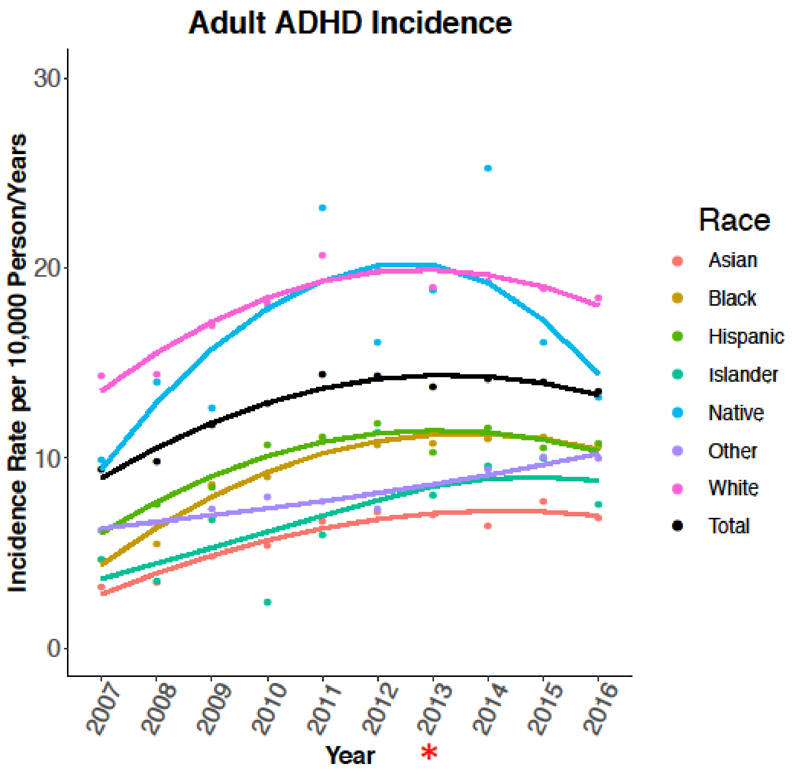

Figure Legend: Incidence (per 10,000 person-years) rates for adult ADHD by year at KPNC from 2007 to 2016. *DSM-5 published in 2013.

© 2019 Chung W et al. *JAMA Network Open.*
